# Combination of external fixation using digital six-axis fixator and internal fixation to treat severe complex knee deformity

**DOI:** 10.1186/s13018-023-03530-0

**Published:** 2023-01-27

**Authors:** Shu-guang Liu, Deng-jie Yu, Hui Li, Michael Opoku, Jun Li, Bao-gang Zhang, Yu-sheng Li, Feng Qiao

**Affiliations:** 1 Department of Joint Surgery, Honghui Hospital, Xi’an Jiao Tong University, Xi’an, Shaan Xi China; 2 Orthopedic Department of Integrated Traditional Chinese and Western Medicine, Honghui Hospital, Xi’an Jiao Tong University, No. 555, Youyi East Road, Nanshaomen, Beilin District, Xi’an City, 710054 Shaan Xi China; 3 Department of Orthopedics, Xiangya Hospital Central South University, No. 87 Xiangya Road, Kaifu District, Changsha City, 410008 Hunan China

**Keywords:** Knee, Osteotomy, Valgus, Varus, Q spatial fixator (QSF)

## Abstract

**Background:**

Severe knee valgus/varus or complex multiplanar deformities are common in clinic. If not corrected in time, cartilage wear will be aggravated and initiate the osteoarthritis due to lower limb malalignment. Internal fixation is unable to correct severe complex deformities, especially when combined with lower limb discrepancy (LLD). Based on the self-designed digital six-axis external fixator Q spatial fixator (QSF), which can correct complex multiplanar deformities without changing structures, accuracy of correction can be improved significantly.

**Methods:**

This retrospective study included 24 patients who suffered from complex knee deformity with LLD treated by QSF and internal fixation at our institution from January 2018 to February 2021. All patients had a closing wedge distal femoral osteotomy with internal fixation for immediate correction and high tibia osteotomy with QSF fixation for postoperative progressive correction. Data of correction prescriptions were computed by software from postoperative CT scans.

**Results:**

Mean discrepancy length of operative side was 2.39 ± 1.04 cm (range 0.9–4.4 cm) preoperatively. The mean difference of lower limb was 0.32 ± 0.13 cm (range 0.11–0.58 cm) postoperatively. The length of limb correction had significant difference (*p* < 0.05). The mean MAD and HKA decreased significantly (*p* < 0.05), and the mean MPTA and LDFA increased significantly (*p* < 0.05). There were significant increase (*p* < 0.05) in the AKSS-O, AKSS-F and Tegner Activity Score. The lower limb alignment was corrected (*p* < 0.05). The mean time of removing external fixator was 112.8 ± 17.9 days (range 83–147 days).

**Conclusions:**

Complex knee deformity with LLD can be treated by six-axis external fixator with internal fixation without total knee arthroplasty. Lower limb malalignment and discrepancy can be corrected precisely and effectively by this approach.

## Background

**Fig. 1:**
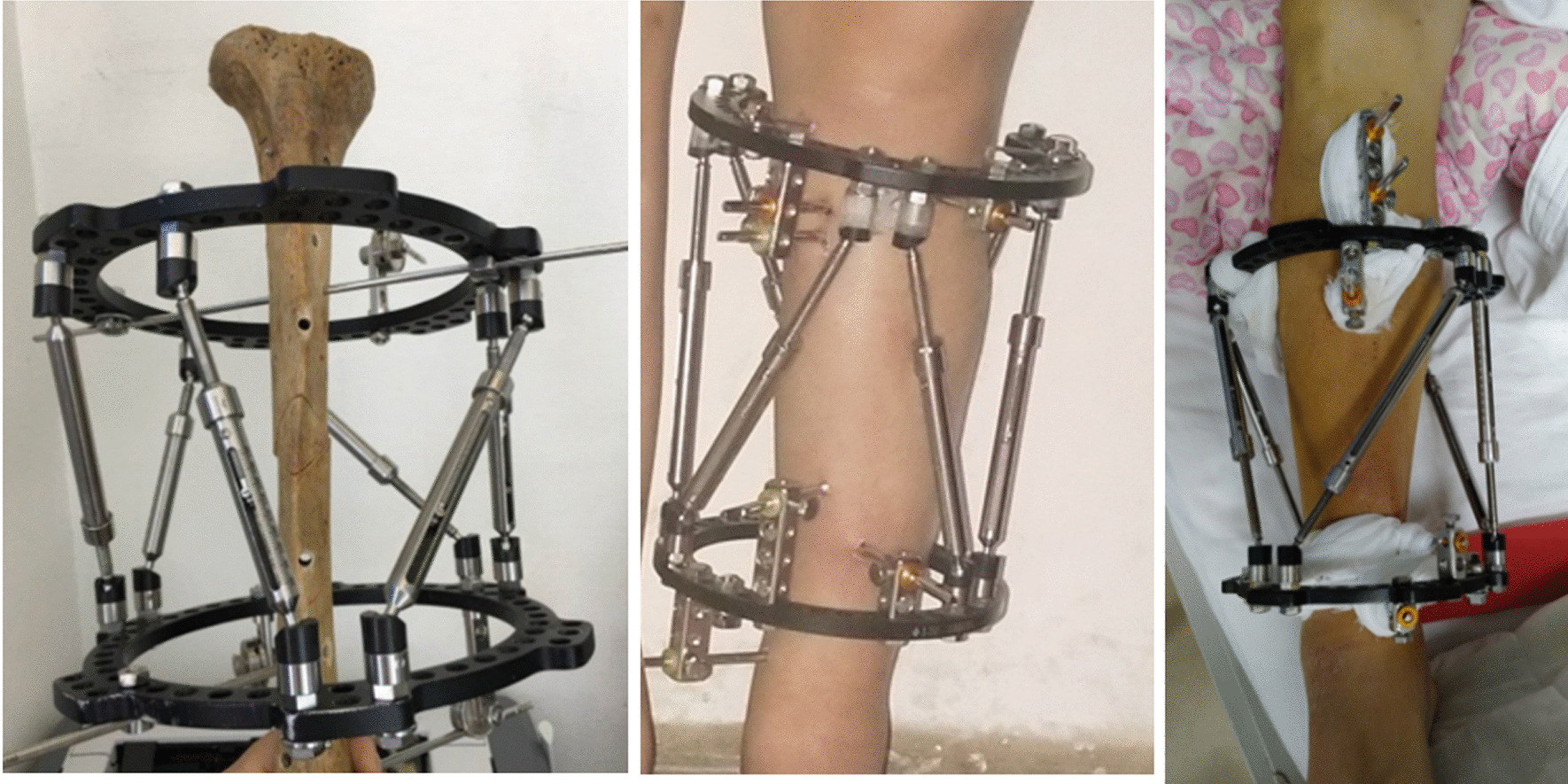
The picture of six-axis Q spatial frame (QSF). It is constructed by two rings and 6 struts. There is a nut on the strut to be screwed to lengthen or shorten the strut. Two kinds of pins are used which are Kirschner pins and Schanz pins. Pins are fixed onto the rings by clamps and conjunction parts. The nut has six facets. The lengths of strut will change 1 mm when the nut is screwed one round

Complex knee deformity with lower limb discrepancy (LLD) is always caused by congenital factors or injury during the period of epiphyseal development. They both affect the lower limb alignment and lead to hip, knee or other severe problems. Because of the deformity of lower limb alignment, the knee compartment is relatively stressed out and degeneration becomes faster [1]. When the degeneration reaches a certain extent, it will only be treated by knee replacement to recover. Distal femoral osteotomy (DFO) is the major surgical approach to treat knee valgus/varus and early osteoarthritis, which can treat malalignment and avoid total joint replacement [2]. LLD can be one of the associated symptoms of knee valgus/varus and can lead to the change of gait. Eventually, the spine will be involved [3]. For simple LLD, Ilizarov external fixator can provide a stable fixation and promote natural bone growth [4]. But for LLD patients accompanied with knee deformity, it is hard to treat by Ilizarov external fixator. Taylor spatial frame (TSF) is a promising method in multiplanar deformities correction and limb lengthening [5, 6]. However, the TSF is hard to operate and the ring is required to be as perpendicular to the tibia as possible. To provide an easy and practical external fixator in the correction of LLD with knee deformity, we designed a digital six-axis external fixator of shape letter Q. Due to the special shape and its designer Prof. Qiao, the fixator was named Q spatial fixator (QSF) (Fig. 1). Depending on the data obtained from computed tomography (CT) scan and the structural optimization, QSF is easier to operate and study, as well as more precise.

## Methods

This study included 24 patients (25 knees) who were treated with the QSF and internal fixation at our department for complex knee deformity from January 2018 to February 2021. Under the assistance of a self-designed angle adjustable osteotomy guide (AAOG) [7], all patients underwent the distal femoral osteotomy (DFO) as well as the tibia and fibular osteotomy, and then, QSF was mounted to the tibia. Follow-up time after operation was > 6 months (3 months after removing QSF). There were 13 women and 11 men with a mean age of (31.8 ± 11.7) years (range 18–55 years). The mean body mass index (BMI) was (25.1 ± 3.4) kg/m^2^ (range 19.8–31.2 kg/m^2^). Six patients had primary knee valgus, and 18 patients had primary knee varus. There were 9 cases of both femoral and tibia sides discrepancy and 15 cases of tibial sides discrepancy. Two patients underwent internal fixation and QSF fixation at both lower limbs. Five patients underwent internal fixation and QSF fixation at one side while internal fixation at another side. All patients did not take surgery before. The study protocol was approved by the Institutional Review Boards and the Ethics Committees. Before surgery, informed consent was obtained from all patients after a full explanation of the therapeutic procedure.

**Fig. 2:**
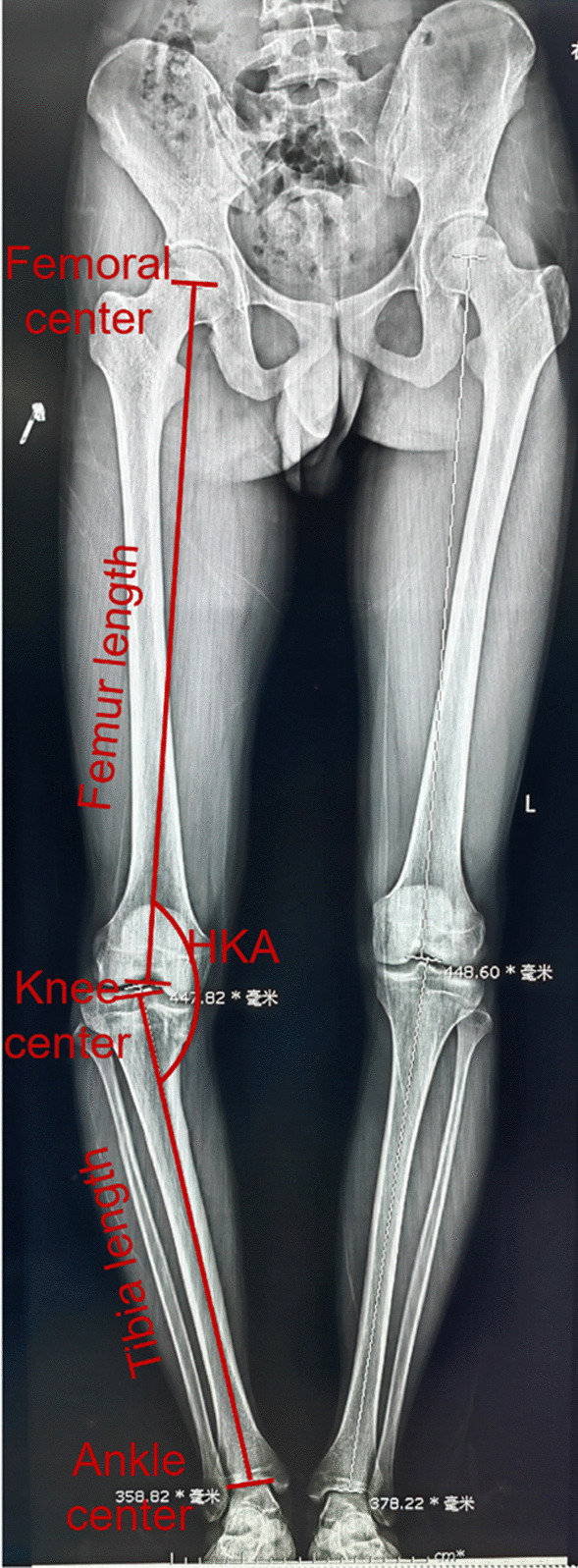
The measurement of lower limb discrepancy and alignment. Lower limb discrepancy was measured by the sum of femur length and tibia length, and the low alignment was measured by HKA

Lower limb discrepancy and alignment were measured by full limb length and hip–knee–ankle angle (HKA) (Fig. 2). HKA was the angle between the mechanical axis of the femur and tibia. To measure the full limb length, the center of the knee was determined first. The length between the center of femoral and the center of the knee was defined as the length of the femur. The length between the center of ankle and the center of the knee was defined as the length of the tibia. The sum of the femur length and the tibia length was defined as the lower limb discrepancy because the aimed HKA angle was 0° [8]. Indications for surgery included: Both femur and tibia sides of lower extremities have the deformities, HKA varus or valgus > 10°. Indication for DFO was: lateral distal femoral angle (LDFA) < 85°. Indications for QSF correction and tibial lengthening included: discrepancy of the lower limb length > 2 cm and medial proximal tibial angle (MPTA) > 5° after DFO simulation.

Related inspections and full-Length weight-bearing radiograph of lower limbs were completed after admission to get preoperative plan. The correction goal was LDFA = 90°. The operative plans were made based on Solidworks™ software. First, we got the height of the osteotomy and the angle of the wedge. Then, we simulated the DFO. Based on the simulated postoperative lower extremities X-ray in the standing position, we measured the difference of the length between the two lower extremities to decide whether the patient should have taken the tibial lengthening and to which extent should the length be extended.

The correction angle of MPTA and the plan of lengthening were decided after operation. Details are as follows: Postoperative spiral CTs were used to calculate the exact difference of HKA and the length of bilateral lower extremities again. Based on the target lower limb alignment and the target lower limb length, we calculated the limb length to be extended and the correction angle by the digital simulated reverse engineering [9] and got the correction plan which was called correction prescription (Table 1).

**Table 1:** Different patients have different correction prescription

Correction prescription Finish adjusting in 14 days. Adjust screws in every morning, noon, night and before going to bed. Adjust poles needed be lengthened first and poles needed be shortened then						
	Anteromedial (No.1)	Medial (No.2)	Posteromedial (No.3)	Posterolateral (No.4)	Lateral (No.5)	Anterolateral (No.6)
Initial length (mm)	157.5	123.0	131.0	178.0	188.0	164.0
Targeted length (mm)	164.9	159.4	168.3	168.3	162.2	149.1
Length adjusted totally (mm)	7.4	36.4	37.3	− 9.7	− 25.8	− 14.9
Length adjusted every day (mm)	0.5	2.6	2.7	− 0.7	− 1.8	− 1.1
Every facet’s length screwed (mm)	0.17	0.17	0.17	0.17	0.17	0.17
How many facets adjusted every day (mm)	3.2	15.6	16.0	− 4.2	− 11.1	− 6.4
How many facets adjusted once (mm)	0.8	3.9	4.0	− 1.0	− 2.8	− 1.6
Day 1 (mm)	158.0	125.6	133.7	177.3	186.2	162.9
Day 2 (mm)	158.6	128.2	136.3	176.6	184.3	161.9
Day 3 (mm)	159.1	130.8	139.0	175.9	182.5	160.8
Day 4 (mm)	159.6	133.4	141.7	175.2	180.6	159.7
Day 5 (mm)	160.1	136.0	144.3	174.5	178.8	158.7
Day 6 (mm)	160.7	138.6	147.0	173.8	176.9	157.6
Day 7 (mm)	161.2	141.2	149.7	173.2	175.1	156.6
Day 8 (mm)	161.7	143.8	152.3	172.5	173.3	155.5
Day 9 (mm)	162.3	146.4	155.0	171.8	171.4	154.4
Day 10 (mm)	162.8	149.0	157.6	171.1	169.6	153.4
Day 11 (mm)	163.3	151.6	150.3	170.4	167.7	152.3
Day 12 (mm)	163.8	154.2	163.0	169.7	165.9	151.2
Day 13 (mm)	164.4	156.8	165.6	169.0	164.0	150.2
Day 14 (mm)	164.9	159.4	168.3	168.3	162.2	149.1

And this chart was given to the patients in Chinese. These patients can finish adjusting in 14 days. Adjust the length of every strut by screwing the nuts in every morning, noon, night and before going to bed. Adjust struts needed be lengthened first and struts needed be shortened then. Every nut has 6 facets. Reading the facets is easier for the patients. When nut was screwed for one round, the length of the rod changed for 1 mm. So, the change of every facet is 0.17 mm. It is not needed to be accurate at the first three times of adjusting every day. Before sleeping, check the length of the rod according to the chart. + means to lengthen. − means to shorten

### Procedures of operation and QSF correction

The procedure for operation was performed with the patient under the supine position and performed combined general anesthesia, with the affected limb disinfected. Tranexamic acid 1 g was injected intravenously prior to incision. A tourniquet was applied at the thigh. Anteromedial incision of the distal femur was utilized, and the medial cortex of the distal femur was exposed. DFO was carried out with the assistance of distal femoral AAOG, and the guide sleeve of adjusted AAOG was placed close to the medial cortex of the distal femur. Along the sleeve, 3 guide pins were placed proximally and distally. Then, we took out the AAOG and the Kirschner wire. Next, we placed the oscillating saw close to the Kirschner wire to cut while protecting the surrounding soft tissue. After completion of the osteotomy, the osteotome was cleaned and the bone block was removed. We then confirmed that the front and back bones were up to the hinge point and then slowly applied varus force until the medial cortex was closed. The patient was temporarily fixed with 2 Kirschner wires and then fixed by AO Tomofix plate (Synthes GmbH, Switzerland). After completion of fixation, the wound was filled with Tranexamic acid gauze and the tourniquet was loosed. Then compression hemostasis for at least 5 min. After no active bleeding was observed, we placed the drainage tube inside and closed the wound by layers.

An approximate 3-cm longitudinal incision was made over the lateral skin of the middle distal fibula to perform fibular osteotomy. The decision of whether to remove a section of the fibula depended on the degree of tibia deformity. Then medial and lateral longitudinal skin incisions were centered over the deformity of the proximal tibia, approximately 1.5 cm in length. The periosteum was cut and separated, and minimally invasive tibia osteotomy (did not cut off completely in order to mount external fixator easily) was performed. The QSF was preconstructed based on the shape of deformity and then the assembled fixator was mounted to the affected limb. Next, we adjusted the locations of rings to make the proximal and distal rings vertical to the anatomical axis of the fixed bone segment, and located in the center of the limb. The distances between rings and the surrounding skin were almost similar (approximately 2–3 cm). After the deformity was corrected, the two rings were basically parallel and appeared beautiful.

C ring was connected, surrounding the tibia proximally in order not to affect the flexion of the knee. Two 2.0-mm Kirschner pins were inserted from posterolateral to anteromedial and from anterolateral to posteromedial, respectively, to fix the distal ring, which was positioned at the distal osteotomy and parallel to the joint line of the ankle. Stretch was done by these two 2.0-mm bilateral external fixator Kirschner pins. Then, connect one or two conjunction parts with holes and 2 Schanz pins were used to fix the distal ring. And Schanz pins were needed for bi-cortical fixation and unilateral external fixation. We checked again to make sure the proximal ring was centered and was not affecting the knee flexion. Then, connect two conjunction parts and 4 Schanz pins were used to fix the proximal ring.

We confirmed whether the lengths of fixation pins were appropriate by fluoroscopic examination. Then, the lengths of 6 connecting struts (initial length) were recorded. An osteotome was used to cut/twist the two rings to allow room for the bone to be cut completely. Then, the 6 connecting struts were restored to their original lengths. The bone surface at the osteotomy site was in good alignment. The wound was then sutured, and the pin sites was fixed. The operation ended successfully.

CT examinations were taken immediately after surgery. Data of the osteotomy site and external fixator were input into software and calculated. We got the length data of each connecting strut of the expected length after the limb was extended, then compared with the raw data and finally calculated the length of each connecting strut to be extended. According to the CT data, we computed the QSF correction prescription by the supporting software and started pulling for bone regeneration according to the correction prescription after 1 week of surgery and pulling speed was 0.7–1.0 mm/d. After discharged, patients should adjust the QSF following the correction prescription. He struts were adjusted in the morning, noon, afternoon and evening, respectively, to meet the length to be extended. After adjusting and fixing the struts, patients were able to stand immediately and walked with assistance. We took lower limb full-length weight-bearing X-ray after correction finished. Then, we made the decision that whether fine turning was needed depended on the result. Adjusted until lower limb alignment was satisfactory, we locked the connecting struts. We took X-ray re-examination monthly. After the osteotomy site recovered, remove the external fixator.

X-ray re-examinations were taken once a month after surgery. After 3.5–5 months of surgery, when the calluses had formed apparently on the osteotomy sites, the screw nuts of each connecting strut were loosed. Patients continued a week walking exercise before the external fixators were removed, and walking under the protection of orthosis after the removal of the external fixators. The orthosis was removed after 2 weeks, and they began to walk normally. Zippers were installed on the sides of the trousers of the patients to facilitate their wearing and taking off.

Outpatient follow-up was taken around 3 months after the removal of the external fixators. Full-length radiographs of both lower extremities in standing position were taken, and the HKA was measured, as well as the MAD, MPTA, LDAF and LDD. Knee joints were assessed by American Knee Society Objective Score (AKSS-O), American Knee Society Functional Score (AKSS-F) and Tegner Activity Score. At the same time, we recorded the occurrence of complications.

### Statistical analysis

The data were analyzed by SPSS version 25.0 for Windows (SPSS Inc., Chicago, IL, USA). Continuous variables with normal distributions were presented as *x* ± *s* and were analyzed by paired samples *T* tests; non-normal variables were reported as median *M*(*Q*_R_) and were analyzed by Pearson *χ*^2^ test. Statistically, significant difference was set at *p* < 0.05.

## Results

Mean discrepancy length of operative side was 2.39 ± 1.04 cm (range 0.9–4.4 cm) preoperatively. The mean difference of lower limb was 0.32 ± 0.13 cm (range 0.11–0.58 cm) postoperatively. The length of limb correction had significant difference (*p* < 0.05). The mean HKA was 15.28 ± 6.05° (range − 19.2°–29°) preoperatively and 1.95 ± 0.94° (range − 2.5° to 4.1°) postoperatively. The lower limb alignment was corrected significantly (*p* < 0.05). The mean time for the removal of the external fixator was 112.8 ± 17.9 days (range 83–147 days).

All patients were followed for more than 3 months after removing the external fixator and the total follow-up, which were totally more than 6 months. MAD decreased from (51.27 ± 20.72) mm (range 26.2–112.0) to (5.59 ± 3.68) mm (range 2–15.1 mm). MPTA increased from (73.23 ± 11.41°) (range 50.40–96.3) to (87.38 ± 1.68°) (range 82.20°–90°). LDFA increased from (79.10 ± 3.85°) (range 70.70–84.3) to (88.11 ± 1.25°) (range 85.60°–90.5°). AKSS-O improved from (71.15 ± 12.75) points (range 50–95points) to (94.81 ± 5.74) points (range 80–100 points) (t = 13.735, *P* = 0.000). AKSS-F was improved from (62.92 ± 11.38) points (range 35–77 points) to (90.27 ± 5.46) points (range 78–100 points) (t = − 16.201, *P* = 0.000). Tegner Activity Score improved from (2.81 ± 1.47) points (range 1–6 points) to (6.19 ± 1.79) points (range 2–9 points) (t = 17.558, *P* = 0.000). All differences had statistical significance when *p* < 0.05. The knee function and motor function were improved significantly (Table 2).

**Table 2:** The MAD, MPTA, LDAF, LLD and HKA were measured on the X-ray films

	Mean ± SD	Minimum	Maximum	Paired samples *t* test	
				*t*	*p*
MAD (mm, *n* = 26)					
Pre	51.27 ± 20.72	26.20	112.00	-11.382	0.00
Post	5.59 ± 3.68	2.00	15.10		
MPTA (°, * n* = 26)					
Pre	73.23 ± 11.41	50.40	96.30	6.596	0.00
Post	87.38 ± 1.68	82.20	90.00		
LDFA (°, * n* = 26)					
Pre	79.10 ± 3.85	70.70	84.30	11.378	0.00
Post	88.11 ± 1.25	85.60	90.50		
LLD (mm, * n* = 24)					
Pre	2.39 ± 1.04	0.90	4.40	-9.666	0.00
Post	0.32 ± 0.13	0.11	0.58		
HKA (°, * n* = 26)					
Pre	15.28 ± 6.05	5.30	29.00	11.323	0.00
Post	1.95 ± 0.94	0.00	4.10		
AKSS-F (*n* = 26)					
Pre	62.92 ± 11.38	35.00	77.00	16.201	0.00
Post	90.27 ± 5.46	78.00	100.00		
AKSS-O (*n* = 26)					
Pre	71.15 ± 12.75	50.00	95.00	13.735	0.00
Post	94.81 ± 5.74	80.00	100.00		
Tegner (*n* = 26)					
Pre	2.81 ± 1.47	1.00	6.00	17.558	0.00
Post	6.19 ± 1.79	2.00	9.00		

The AKSS-F, AKSS-O and Tegner score were recorded from patients

*MAD* mechanical axis deviation, *MPTA* medial proximal tibial angle, *LDFA* lateral distal femur angle, *LLD* Leg length discrepancy, *HKA* hip–knee–ankle angle, *AKSS-F* American Knee Society Functional Score, *AKSS-O* American Knee Society Objective Score; Paired samples *t* test was used

**Fig. 3:**
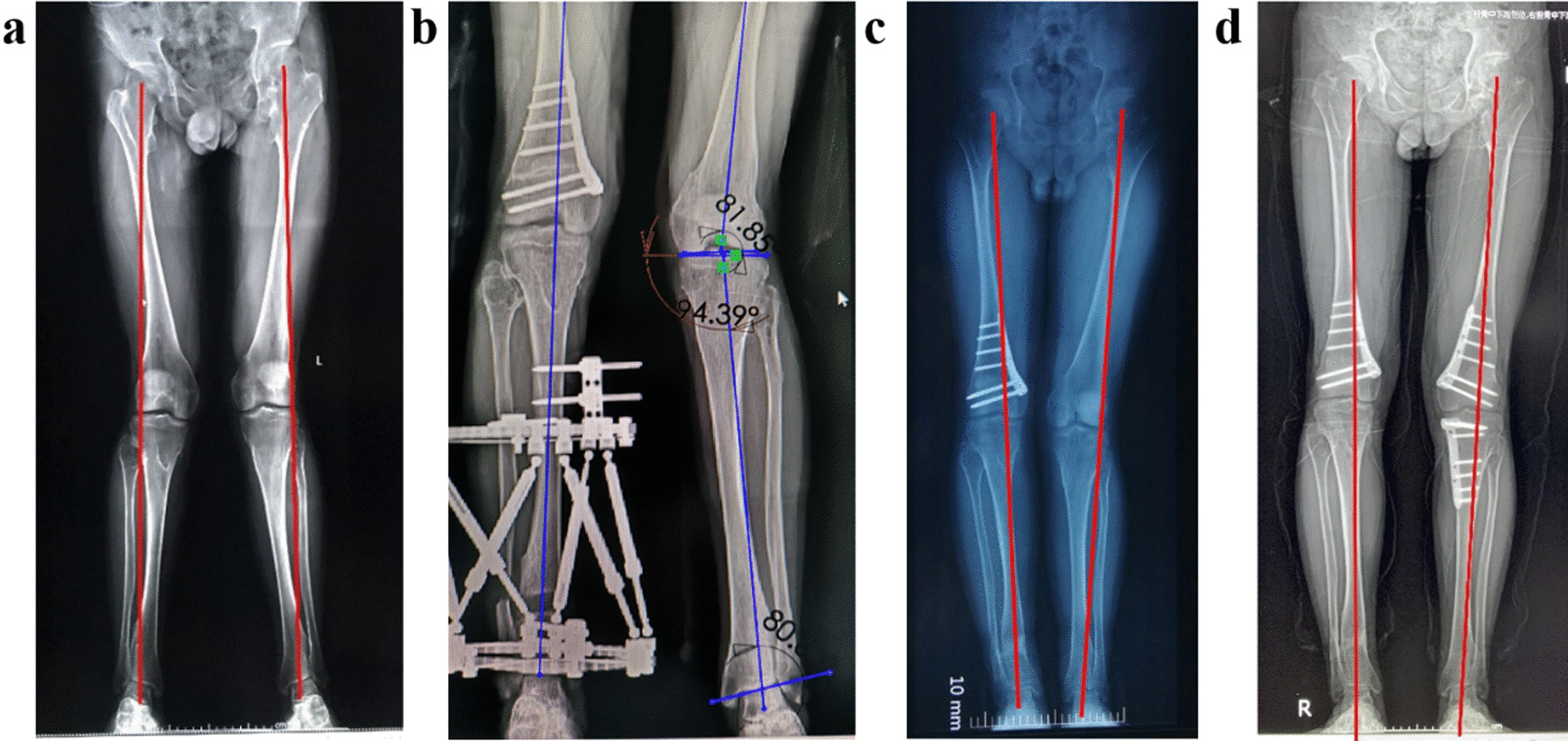
An 18-year-old patient had bilateral knee and malleolus valgus because of multiple osteochondroma. The right lower limb was 3.3 cm shorter than the left. **a** Full-length radiograph before operation. **b** X-ray after operation shows the deformity has been corrected. **c** Left lower limb surgery will be taken after 1 year. **d** Osteotomy and internal fixation at the left side. Re-examination X-ray shows the osteotomy site healed well. The lower limb alignment of right limb and the LLD is near normal

**Fig. 4:**
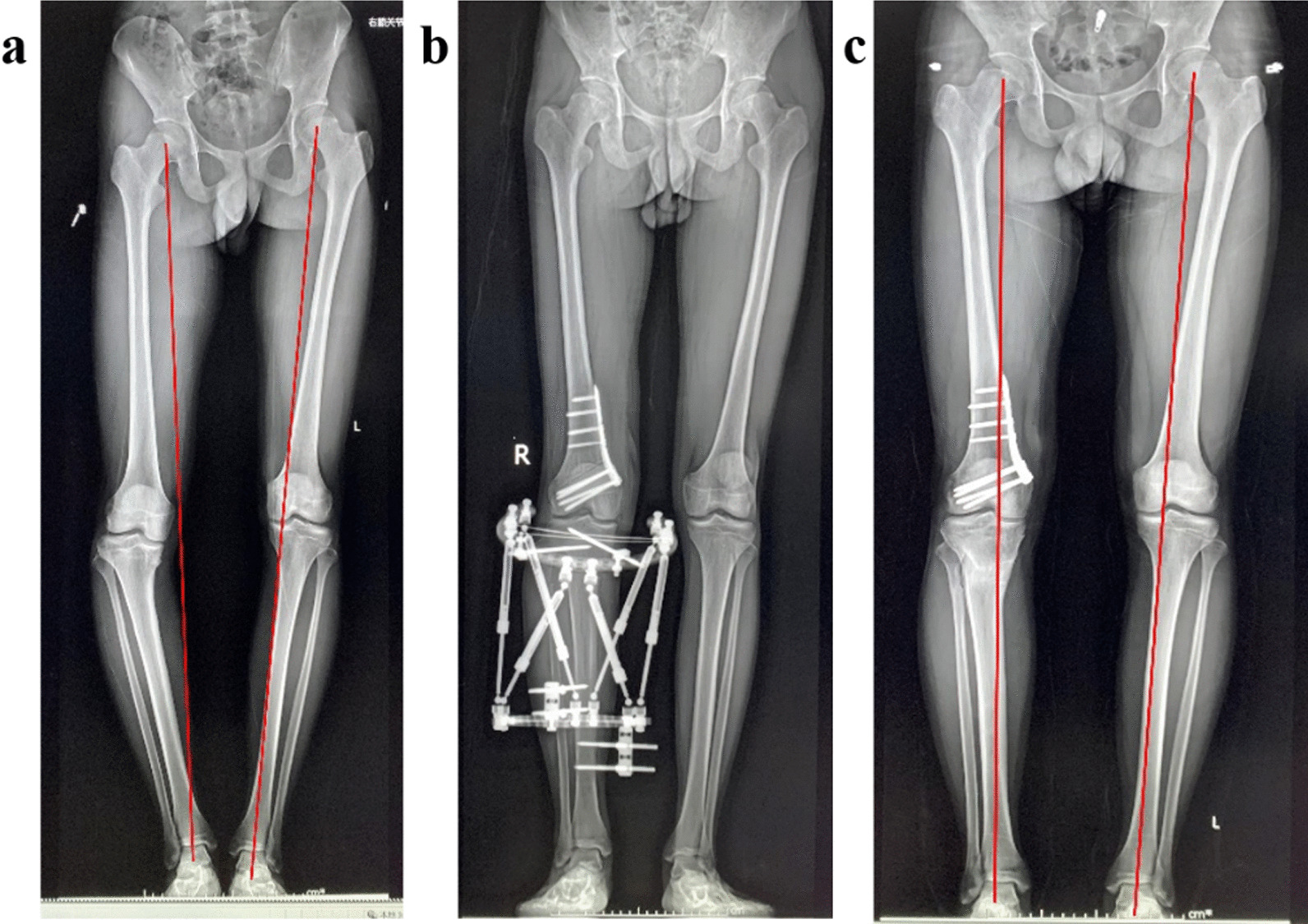
An 18-year-old patient had right varus knee because of Blount disease. The right lower limb was 2 cm shorter than the left. **a** Full-length radiograph before operation. **b** X-ray after operation shows DFO was, and QSF was applied. Re-examination X-ray shows the osteotomy site healed well, and the QSF was removed. The lower limb alignment of right limb and the LLD is near normal

Obvious continuous callus was formed at the osteotomy site. None of the patients said it had an obvious negative impact on quality of life. There were no complications such as nonunion and pin sites infection. No infection was occurred with only several cases which had small amount of pin sites secretors. For these cases, it was recovered naturally after removing the QSF with regular disinfection. Re-examination was done 8 weeks after the removal of the external fixator. All patients returned to normal lives and no fractures occurred. Typical cases are shown in Figs. 3 and 4.

## Discussion

The abnormal excessive pressure imposed on the knee compartment is associated with the initiation and progression of osteoarthritis [10]. Knee valgus/varus deformity combined with LLD is common and hard to treat. Because it is a complex and multiplanar deformity, only internal fixation is unable to correct this complex deformity. While lengthening the limb, the rotation of the affected limb also needs to be corrected. Compared with traditional treatments, hexapod fixator can distract bone progressively to correct deformities from different planes and has been widely used in deformity correction [11, 12]. Based on the hexapod principle, we developed a digital six-axis fixator. The results of our study demonstrated that the combination of our self-designed QSF and internal fixator was an effective treatment in patients with severe knee deformities combined with LLD. Lower limb length and alignment of patients were corrected to normal range. All patients had significant improvement in their AKSS-O, AKSS-F and Tegner Activity Score.

Distal femoral osteotomy (DFO) is a surgical method to treat osteoarthritis and knee deformities. Particularly, when valgus deformity is > 12° or joint line obliquity > 10°, DFO is the best choice for correction [13]. Though total knee arthroplasty has a good correction effect and long-term result, its high cost and great harm are an obstacle for many patients [14]. Also, knee arthroplasty is not suitable for young patients. In our research, the average age of our patients was only 33 years old and the oldest patient was 55 years old. Therefore, we used DFO for the deformity correction. The correction accuracy is significantly associated with the outcome and survival time for patients. Felson et al. demonstrated that the outcome of valgus alignment depends on the valgus mechanical axis. When mechanical axis ranges from 1.1° to 3° valgus, the risk of OA progression increases. And valgus alignment > 3° is associated with OA incidence [15]. Some studies showed that the accuracy of internal fixation only was not perfect. Overcorrection and undercorrection happened sometimes [16]. Internal fixation also has the risk of rotational malalignment [17]. External fixation can compensate the correct deviation of internal fixation by changing MPTA. But conventional Ilizarov external fixator is not proper for multiplanar deformities correction because it was major for lengthening and axial correction. Hexapod external fixator has been proven to be effective in multiplanar deformities correction with high precision [18, 19].

LLD is very common in public. 90% of the normal people are different in lower limb length, and the average discrepancy was found to be 5.2 mm [20]. Most time the length discrepancy does not affect the function and gait. It will be compensated by mild passive structural changes. But those compensations are not enough for the significant length discrepancy [21]. Gait analysis indicates that when the length discrepancy is > 1 cm, the gait will be asymmetry [22]. A cohort study indicated that the length discrepancy > 2 cm was associated with the knee OA progression and patients will have clinical symptoms in standing [23]. Conservative treatment like shoe lift is recommended by some guidelines [22]. But a systematic review showed that the effect of shoe lift to improve function and relieve pain is uncertain because the evidence of quality was low [24]. Therefore, 2 cm is a generally accepted indication for surgery of LLD [25, 26]. External fixation has long been the major method to treat LLD since the invention of Ilizarov external fixator [27]. For patients have severe knee deformity with LDD, hexapod external fixator can treat both LDD and deformity. In our study, the least discrepancy was only 0.9 mm. The only discrepancy did not have to correct. But the MPTA of this patient was 74.2°, and the deviation may be caused by the metaphyseal deformity instead of the bone development. During the process of correction, an external fixator can adjust the length at the same time to make the limbs tend to be equal in length. Infection risk should also be taken into consideration in the application of internal fixation with QSF. In our study, no infections occurred and only some cases had a small amount of pin sites secretors. Nolte et al. revealed there was no difference between pin sites debridement and simply disinfection in external fixation [28]. Therefore, patients with pin sites secretors were asked to simply disinfection regularly. After removed the pin, they were recovered naturally.

The reasons for the indications of our study (LLD and MPTA difference) were: those who had length discrepancy without MPTA difference, DFO was not essential, and Ilizarov external fixator was effective in limb lengthening [29], for those who had MPTA difference without LLD, and internal fixation was sufficient to meet correction needs. All the patients took osteotomy at the femoral sites. Because the distal lateral femoral angle (DLFA) of some patients was small, osteotomy at the tibia site led to the joint line oblique while at the femoral site did not alter it [30].

The limitation of our study is the small number of patients. Although all our patients achieved satisfactory results, we did not observe any complications like nonunion and needle tract infections of the external fixator [31]. We should enlarge our samples and assess the safety of both our surgery method and external fixator. The data for correction were from CT scans and computer analyses before surgery. To save cost, we choose X-ray to measure the limb length after surgery and in re-examination. So the real results were better than measured by X-ray.

## Conclusions

Knee valgus combined with LLD can be treated by QSF with internal fixation without the need for total knee arthroplasty. Lower limb malalignment and discrepancy can be corrected precisely and effectively by this approach. Patients can walk soon after the procedure and avoid undergoing knee arthroplasty in the advanced stage of OA.

## Data Availability

All the data will be available upon motivated request to the corresponding author of the present paper.
